# Crystal structures of dimethyl 5-iodo­iso­phthal­ate and dimethyl 5-ethynyl­iso­phthal­ate

**DOI:** 10.1107/S205698901800912X

**Published:** 2018-07-13

**Authors:** Ines Hauptvogel, Wilhelm Seichter, Edwin Weber

**Affiliations:** a TU Bergakademie Freiberg, Leipziger Str. 29, D-09596 Freiberg/Sachsen, Germany

**Keywords:** crystal structure, 5-substituted dimethyl isophthalates, I⋯O=C inter­action, C—H⋯O and C—H⋯I hydrogen bonding, π–π stacking

## Abstract

In dimethyl 5-iodo­isophthalate, C_10_H_9_IO_4_, (I), the planes through the methyl carboxyl­ate moieties are tilted with respect to the benzene ring, whereas the mol­ecular framework of dimethyl 5-ethynylisophthalate, C_12_H_10_O_4_, (II), is perfectly planar. The crystal structure of (I) is stabilized by a three-dimensional supra­molecular network comprising C—H⋯O=C hydrogen bonds, as well as I⋯O=C inter­actions. In the crystal of (II), the mol­ecules are connected *via* C—H_ethyn­yl_⋯O=C hydrogen bonds to infinite strands. Moreover, π–π arene stacking inter­actions connect the mol­ecular chains into two-dimensional supra­molecular aggregates.

## Abstract 2

The 5-iodo- and 5-ethynyl-substituted dimethyl isophthalates show mol­ecular frameworks with methyl carboxyl­ate moieties being tilted or perfectly planar with respect to the benzene ring, respectively. Crystal structures feature a three- or two-dimensional supra­molecular aggregation in the iodo and ethynyl derivatives, respectively, supported by C—H⋯I and C—H⋯O hydrogen bonding as well as I⋯O and π–π inter­actions.

## Chemical context   

In recent years, the design of solid porous framework materials (MacGillivray, 2010 ▸; Furukawa *et al.*, 2013 ▸; Eddaoudi *et al.*, 2015 ▸) has become a very important topic in the field of supra­molecular crystal engineering (Desiraju *et al.*, 2011 ▸). Associated with it, so-called linker mol­ecules featuring a geometrically rigid structure frequently being of linear, trigonal or tetra­hedral shape and having carb­oxy­lic acid functions as terminal groups play a key role in building such systems (Lin *et al.*, 2006 ▸; Hausdorf *et al.*, 2009 ▸; Zheng *et al.*, 2010 ▸). In the course of the synthesis of the respective linkers, the title compounds (I) and (II), both being 5-substituted dimethyl isophthalates, are much used inter­mediates. However, these compounds are not only synthetically significant but also show inter­esting structures in the crystalline state, as demonstrated herein.
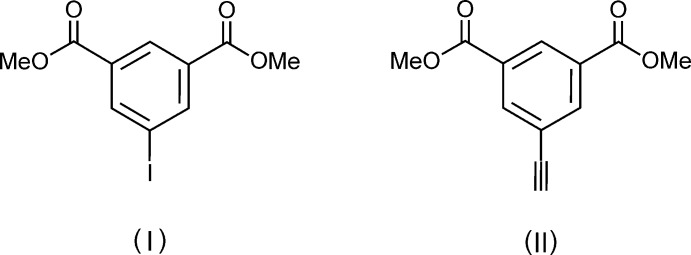


## Structural commentary   

**Figure 1:**
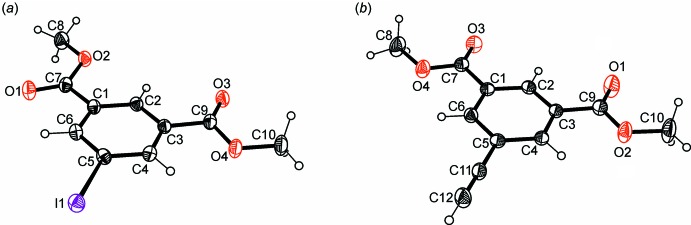
Perspective view of the mol­ecular structures of the title compounds, (*a*) (I) and (*b*) (II), with atom labelling. Anisotropic displacement ellipsoids are drawn at the 40% probability level.

The mol­ecular structures of the title compounds, (I) and (II), are illustrated in Fig. 1 ▸ *a* and 1*b*, respectively. Taking into account experimental error, the bond distances within the isophthalate framework agree well with those found in the crystal structure of dimethyl isophthalate (Gallagher, 2012 ▸). Compound (I) crystallizes in the ortho­rhom­bic space group *Pna*2_1_ with one mol­ecule in the asymmetric unit. The mol­ecule adopts a twisted conformation with the mean planes defined by the methyl carboxyl­ate moieties inclined at angles of 12.6 (2) and 6.0 (2)° with respect to the plane of the benzene ring. Compound (II) crystallizes in the ortho­rhom­bic space group *Pnma* with the mol­ecule located on a symmetry plane, *i.e*. the mol­ecule is perfectly planar. However, the mol­ecule adopts approximate *C*_2*v*_ symmetry with the atoms C2, C5, C11 and C12 lying on a non-crystallographic bis­ecting symmetry plane.

## Supra­molecular features   

**Figure 2:**
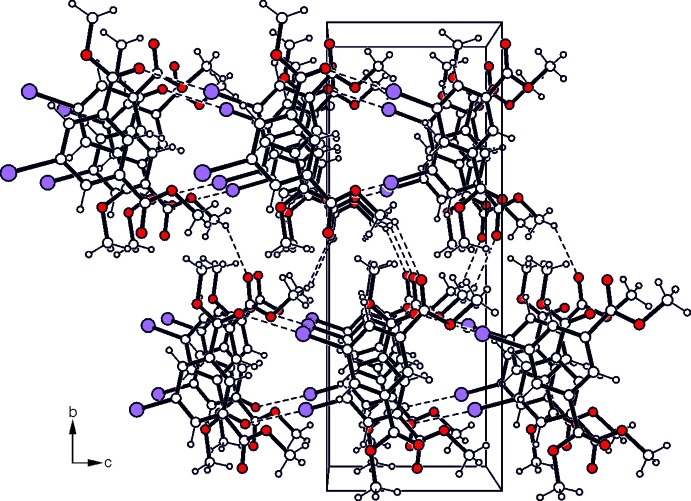
Packing diagram of compound (I) viewed down the *a* axis. Dashed lines represent hydrogen-bonding inter­actions.

**Figure 3:**
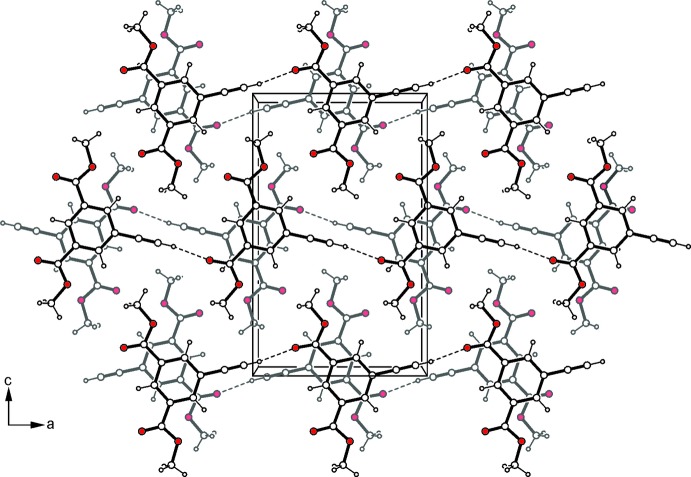
Packing excerpt of compound (II) viewed down the *b* axis. Dashed lines represent hydrogen-bonding inter­actions.

Infinite strands with the mol­ecules connected *via* I⋯O=C interactions [I1⋯O3—C9(*x* − 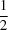, *y* + 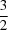, *z* − 1; *D*⋯*A* = 3.129 (2) (Desiraju & Steiner, 1999 ▸) (Politzer *et al.* 2007 ▸; Desiraju *et al.*, 2013 ▸), represent the basic supra­molecular aggregates of the crystal structure of (I). Association of the mol­ecular strands by C—H⋯O=C type hydrogen bonds (Table 1 ▸) (Desiraju & Steiner, 1999 ▸) and π–π stacking inter­actions [centroid–centroid distance = 4.149 (2) Å] (Tiekink & Zukerman-Schpector, 2012 ▸) generate a three-dimensional supra­molecular network (Fig. 2 ▸). In the crystal structure of (II), the mol­ecules are connected *via* C_ethyn­yl_—H⋯O=C bonds (Table 2 ▸) into infinite strands, which are further arranged into mol­ecular sheets that extend parallel to the *ac* plane (Fig. 3 ▸). Furthermore, π–π arene inter­actions with a centroid–centroid distance of 3.566 (1) Å and a slippage of 1.325 Å between the inter­acting aromatic rings stabilize the crystal structure along the stacking axis of the mol­ecular sheets.

## Database survey   

The search in the Cambridge Structural Database (CSD, Version 5.38, update May 2017; Groom *et al.*, 2016 ▸) for *meta*-substituted derivatives of dimethyl isophthalate excluding their metal complexes, solvates and salts gave 18 hits. None of these compounds represents a 5-halogen- and 5-alkynyl-substituted dimethyl isophalate. The parent compound, dimethyl isophthalate (CSD refcode GOHRUS; Gallagher & Mocilac, 2012 ▸) crystallizes in space group *Pna*2_1_ with two conformationally similar mol­ecules in the asymmetric unit. The independent mol­ecules participate in different ways in non-covalent bonding. One of them is involved in the formation of linear strands with the mol­ecules connected by C—H_ar­yl_⋯O=C bonds. Inter­strand association is accomplished by π–π arene stacking. Mol­ecules related by the twofold screw axis are also linked *via* C—H_ar­yl_⋯O=C bonding to form helical strands. In addition, these strands are stabilized by π–π stacking forces.

## Synthesis and crystallization   

Compounds (I) and (II) were synthesized following literature procedures. This involves a diazo­tization/iodination reaction of dimethyl 5-amino­isophthalate (Mazik & König, 2006 ▸) to give compound (I). Subsequent reaction of (I) with 2-methyl­but-3-yne-2-ol (MEBYNOL) using a Pd-catalysed Sonogashira coupling procedure (Doucet & Hierso, 2007 ▸; Rafael & Carmen, 2007 ▸) yielded the corresponding blocked acetyl­enic diester as an inter­mediate (Hauptvogel *et al.*, 2011 ▸). Removal of the 2-hy­droxy­propyl blocking group was undertaken using sodium hydride in toluene and quenching with water to result in the title compound (II) (Havens & Hergenrother, 1985 ▸; Hauptvogel *et al.*, 2011 ▸).

## Refinement   

Crystal data, data collection and structure refinement details are summarized in Table 3 ▸. Hydrogen atoms were positioned geometrically and refined using a riding model with C—H distances of 0.94–0.98 Å and *U*_iso_(H) = 1.5*U*_eq_(C-meth­yl) or *U*_iso_(H) = 1.2*U*_eq_(C) for other H atoms.

## Supplementary Material

Crystal structure: contains datablock(s) I, II, global. DOI: 10.1107/S205698901800912X/zl2732sup1.cif

Structure factors: contains datablock(s) I. DOI: 10.1107/S205698901800912X/zl2732Isup4.hkl

Structure factors: contains datablock(s) II. DOI: 10.1107/S205698901800912X/zl2732IIsup5.hkl

Supporting information file. DOI: 10.1107/S205698901800912X/zl2732Isup4.cml

Supporting information file. DOI: 10.1107/S205698901800912X/zl2732IIsup5.cml

CCDC references: 780476, 780475

Additional supporting information:  crystallographic information; 3D view; checkCIF report

## Figures and Tables

**Table 1:** Hydrogen-bond geometry (Å, °) for (I)

*D*—H⋯*A*	*D*—H	H⋯*A*	*D*⋯*A*	*D*—H⋯*A*
C8—H8*A*⋯O1^i^	0.98	2.55	3.257 (4)	129

Symmetry code: (i) 
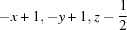
.

**Table 2:** Hydrogen-bond geometry (Å, °) for (II)

*D*—H⋯*A*	*D*—H	H⋯*A*	*D*⋯*A*	*D*—H⋯*A*
C12—H12⋯O1^i^	0.94	2.29	3.223 (1)	172

Symmetry code: (i) 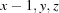.

**Table 3:** Experimental details

	(I)	(II)
Crystal data		
Chemical formula	C_10_H_9_IO_4_	C_12_H_10_O_4_
*M*_r_	320.07	218.20
Crystal system, space group	Orthorhombic, *P* *n* *a*2_1_	Orthorhombic, *P* *n* *m* *a*
Temperature (K)	143	223
*a*, *b*, *c* (Å)	7.7483 (2), 19.3451 (6), 7.2338 (2)	10.1206 (5), 6.6219 (4), 16.3658 (8)
*V* (Å^3^)	1084.29 (5)	1096.80 (10)
*Z*	4	4
Radiation type	Mo *K*α	Mo *K*α
μ (mm^−1^)	2.94	0.10
Crystal size (mm)	0.30 × 0.22 × 0.15	0.54 × 0.12 × 0.10

Data collection		
Diffractometer	Bruker APEXII CCD area detector	Bruker APEXII CCD area detector
Absorption correction	Multi-scan (*SADABS*; Sheldrick, 2008*a* ▸)	Multi-scan (*SADABS*; Sheldrick, 2008*a* ▸)
*T*_min_, *T*_max_	0.472, 0.666	0.948, 0.990
No. of measured, independent and observed [*I* > 2σ(*I*)] reflections	22794, 2909, 2806	12397, 1292, 932
*R*_int_	0.026	0.033
(sin θ/λ)_max_ (Å^−1^)	0.684	0.638

Refinement		
*R*[*F* ^2^ > 2σ(*F* ^2^)], *wR*(*F* ^2^), *S*	0.015, 0.038, 1.05	0.039, 0.110, 1.03
No. of reflections	2909	1292
No. of parameters	139	87
No. of restraints	1	0
H-atom treatment	H-atom parameters constrained	H-atom parameters constrained
Δρ_max_, Δρ_min_ (e Å^−3^)	0.47, −0.44	0.17, −0.18
Absolute structure	Flack *x* determined using 1255 quotients [(*I* ^+^)−(*I* ^−^)]/[(*I* ^+^)+(*I* ^−^)] (Parsons *et al*., 2013 ▸)	–
Absolute structure parameter	−0.004 (8)	–

Computer programs: *APEX2* and *SAINT* (Bruker, 2014 ▸), *SHELXT* (Sheldrick, 2015*a* ▸), *SHELXL2018* (Sheldrick, 2015*b* ▸), *ORTEP-3 for Windows* (Farrugia, 2012 ▸) and *SHELXTL* (Sheldrick, 2008*b* ▸).

## supplementary crystallographic information

### 1,3-Dimethyl 1-iodocyclohexa-3,5-diene-1,3-dicarboxylate (I) Crystal data

**Table:**

C_10_H_9_IO_4_	*D*_x_ = 1.961 Mg m^−^^3^
*M**_r_* = 320.07	Mo *K*α radiation, λ = 0.71073 Å
Orthorhombic, *P**n**a*2_1_	Cell parameters from 5755 reflections
*a* = 7.7483 (2) Å	θ = 3.0–33.7°
*b* = 19.3451 (6) Å	µ = 2.94 mm^−^^1^
*c* = 7.2338 (2) Å	*T* = 143 K
*V* = 1084.29 (5) Å^3^	Irregular, colourless
*Z* = 4	0.30 × 0.22 × 0.15 mm
*F*(000) = 616	

### 1,3-Dimethyl 1-iodocyclohexa-3,5-diene-1,3-dicarboxylate (I) Data collection

**Table:**

Bruker APEXII CCD area detector diffractometer	2806 reflections with *I* > 2σ(*I*)
φ and ω scans	*R*_int_ = 0.026
Absorption correction: multi-scan (SADABS; Sheldrick, 2008a)	θ_max_ = 29.1°, θ_min_ = 1.1°
*T*_min_ = 0.472, *T*_max_ = 0.666	*h* = −10→10
22794 measured reflections	*k* = −26→26
2909 independent reflections	*l* = −9→9

### 1,3-Dimethyl 1-iodocyclohexa-3,5-diene-1,3-dicarboxylate (I) Refinement

**Table:**

Refinement on *F*^2^	Hydrogen site location: inferred from neighbouring sites
Least-squares matrix: full	H-atom parameters constrained
*R*[*F*^2^ > 2σ(*F*^2^)] = 0.015	*w* = 1/[σ^2^(*F*_o_^2^) + (0.019*P*)^2^ + 0.3689*P*] where *P* = (*F*_o_^2^ + 2*F*_c_^2^)/3
*wR*(*F*^2^) = 0.038	(Δ/σ)_max_ = 0.002
*S* = 1.05	Δρ_max_ = 0.47 e Å^−^^3^
2909 reflections	Δρ_min_ = −0.44 e Å^−^^3^
139 parameters	Absolute structure: Flack *x* determined using 1255 quotients [(*I*^+^)-(*I*^-^)]/[(*I*^+^)+(*I*^-^)] (Parsons et al., 2013)
1 restraint	Absolute structure parameter: −0.004 (8)

### 1,3-Dimethyl 1-iodocyclohexa-3,5-diene-1,3-dicarboxylate (I) Special details

**Table:**

Geometry. All esds (except the esd in the dihedral angle between two l.s. planes) are estimated using the full covariance matrix. The cell esds are taken into account individually in the estimation of esds in distances, angles and torsion angles; correlations between esds in cell parameters are only used when they are defined by crystal symmetry. An approximate (isotropic) treatment of cell esds is used for estimating esds involving l.s. planes.
Refinement. Refined as a 2-component twin.

### 1,3-Dimethyl 1-iodocyclohexa-3,5-diene-1,3-dicarboxylate (I) Fractional atomic coordinates and isotropic or equivalent isotropic displacement parameters (Å^2^)

**Table:**

	*x*	*y*	*z*	*U*_iso_*/*U*_eq_	
I1	0.81504 (2)	0.66115 (2)	0.83115 (6)	0.02386 (5)	
O1	0.5202 (3)	0.54751 (11)	0.1864 (3)	0.0327 (5)	
O2	0.3831 (3)	0.63417 (11)	0.0411 (3)	0.0260 (4)	
O3	0.4971 (3)	0.87789 (10)	0.2029 (3)	0.0281 (5)	
O4	0.6557 (3)	0.89701 (11)	0.4576 (3)	0.0282 (5)	
C1	0.5514 (4)	0.66267 (12)	0.2993 (4)	0.0190 (8)	
C2	0.5347 (3)	0.73272 (14)	0.2585 (4)	0.0198 (5)	
H2	0.479369	0.746965	0.147720	0.024*	
C3	0.5994 (3)	0.78152 (14)	0.3805 (3)	0.0194 (5)	
C4	0.6796 (3)	0.76086 (15)	0.5445 (4)	0.0206 (5)	
H4	0.723931	0.794439	0.627770	0.025*	
C5	0.6940 (3)	0.69088 (15)	0.5850 (4)	0.0207 (5)	
C6	0.6295 (4)	0.64128 (15)	0.4637 (4)	0.0209 (5)	
H6	0.638485	0.593502	0.492478	0.025*	
C7	0.4856 (4)	0.60819 (14)	0.1721 (4)	0.0223 (5)	
C8	0.3221 (5)	0.58545 (18)	−0.0951 (5)	0.0330 (7)	
H8A	0.418544	0.571014	−0.173823	0.049*	
H8B	0.232932	0.607288	−0.171341	0.049*	
H8C	0.273509	0.544937	−0.032593	0.049*	
C9	0.5768 (3)	0.85636 (11)	0.3323 (8)	0.0214 (4)	
C10	0.6363 (5)	0.97101 (16)	0.4293 (5)	0.0338 (7)	
H10A	0.688803	0.984036	0.311034	0.051*	
H10B	0.693716	0.995947	0.529896	0.051*	
H10C	0.513396	0.982919	0.427800	0.051*	

### 1,3-Dimethyl 1-iodocyclohexa-3,5-diene-1,3-dicarboxylate (I) Atomic displacement parameters (Å^2^)

**Table:**

	*U*^11^	*U*^22^	*U*^33^	*U*^12^	*U*^13^	*U*^23^
I1	0.02804 (8)	0.02336 (8)	0.02019 (8)	−0.00006 (6)	−0.00121 (12)	0.00381 (11)
O1	0.0500 (14)	0.0174 (10)	0.0308 (12)	0.0032 (9)	−0.0060 (10)	−0.0006 (9)
O2	0.0300 (11)	0.0187 (10)	0.0293 (11)	0.0017 (8)	−0.0066 (9)	−0.0048 (8)
O3	0.0347 (12)	0.0194 (10)	0.0301 (11)	0.0027 (9)	−0.0082 (9)	0.0019 (8)
O4	0.0382 (12)	0.0165 (9)	0.0299 (11)	−0.0011 (8)	−0.0073 (9)	−0.0004 (9)
C1	0.0216 (11)	0.0183 (10)	0.017 (2)	0.0009 (9)	0.0020 (10)	−0.0004 (9)
C2	0.0196 (12)	0.0190 (12)	0.0209 (11)	0.0022 (10)	0.0042 (10)	0.0019 (10)
C3	0.0191 (11)	0.0183 (11)	0.0208 (13)	0.0013 (9)	0.0025 (9)	0.0012 (8)
C4	0.0217 (13)	0.0191 (13)	0.0211 (12)	−0.0014 (10)	0.0017 (10)	−0.0012 (10)
C5	0.0224 (13)	0.0217 (13)	0.0181 (12)	0.0022 (10)	0.0023 (10)	0.0017 (10)
C6	0.0242 (13)	0.0183 (12)	0.0201 (12)	0.0016 (10)	0.0024 (11)	0.0013 (10)
C7	0.0256 (13)	0.0197 (12)	0.0217 (13)	−0.0011 (10)	0.0035 (11)	−0.0011 (10)
C8	0.0409 (19)	0.0257 (15)	0.0323 (15)	0.0005 (13)	−0.0088 (13)	−0.0075 (13)
C9	0.0218 (10)	0.0173 (9)	0.0250 (10)	−0.0001 (8)	0.0096 (18)	−0.002 (2)
C10	0.0453 (19)	0.0174 (14)	0.0387 (19)	0.0014 (13)	−0.0064 (15)	−0.0021 (12)

### 1,3-Dimethyl 1-iodocyclohexa-3,5-diene-1,3-dicarboxylate (I) Geometric parameters (Å, º)

**Table:**

I1—C5	2.093 (3)	C3—C4	1.398 (4)
O1—C7	1.209 (3)	C3—C9	1.499 (4)
O2—C7	1.335 (4)	C4—C5	1.390 (4)
O2—C8	1.443 (4)	C4—H4	0.9500
O3—C9	1.196 (5)	C5—C6	1.393 (4)
O4—C9	1.347 (5)	C6—H6	0.9500
O4—C10	1.454 (4)	C8—H8A	0.9800
C1—C2	1.393 (3)	C8—H8B	0.9800
C1—C6	1.398 (4)	C8—H8C	0.9800
C1—C7	1.489 (4)	C10—H10A	0.9800
C2—C3	1.386 (4)	C10—H10B	0.9800
C2—H2	0.9500	C10—H10C	0.9800
			
C7—O2—C8	115.7 (2)	C1—C6—H6	120.4
C9—O4—C10	115.7 (3)	O1—C7—O2	124.0 (3)
C2—C1—C6	120.5 (3)	O1—C7—C1	124.0 (3)
C2—C1—C7	121.8 (3)	O2—C7—C1	112.1 (2)
C6—C1—C7	117.7 (2)	O2—C8—H8A	109.5
C3—C2—C1	119.7 (3)	O2—C8—H8B	109.5
C3—C2—H2	120.2	H8A—C8—H8B	109.5
C1—C2—H2	120.2	O2—C8—H8C	109.5
C2—C3—C4	120.4 (3)	H8A—C8—H8C	109.5
C2—C3—C9	117.9 (3)	H8B—C8—H8C	109.5
C4—C3—C9	121.7 (3)	O3—C9—O4	123.9 (2)
C5—C4—C3	119.5 (3)	O3—C9—C3	125.3 (3)
C5—C4—H4	120.2	O4—C9—C3	110.7 (3)
C3—C4—H4	120.2	O4—C10—H10A	109.5
C4—C5—C6	120.6 (3)	O4—C10—H10B	109.5
C4—C5—I1	118.9 (2)	H10A—C10—H10B	109.5
C6—C5—I1	120.5 (2)	O4—C10—H10C	109.5
C5—C6—C1	119.2 (3)	H10A—C10—H10C	109.5
C5—C6—H6	120.4	H10B—C10—H10C	109.5
			
C6—C1—C2—C3	1.3 (4)	C8—O2—C7—O1	−4.1 (4)
C7—C1—C2—C3	−179.4 (2)	C8—O2—C7—C1	176.3 (3)
C1—C2—C3—C4	−0.6 (4)	C2—C1—C7—O1	168.0 (3)
C1—C2—C3—C9	−179.3 (3)	C6—C1—C7—O1	−12.7 (4)
C2—C3—C4—C5	−0.1 (4)	C2—C1—C7—O2	−12.5 (4)
C9—C3—C4—C5	178.5 (3)	C6—C1—C7—O2	166.8 (3)
C3—C4—C5—C6	0.1 (4)	C10—O4—C9—O3	1.1 (5)
C3—C4—C5—I1	179.86 (19)	C10—O4—C9—C3	−177.5 (3)
C4—C5—C6—C1	0.7 (4)	C2—C3—C9—O3	5.3 (5)
I1—C5—C6—C1	−179.1 (2)	C4—C3—C9—O3	−173.3 (3)
C2—C1—C6—C5	−1.3 (4)	C2—C3—C9—O4	−176.1 (3)
C7—C1—C6—C5	179.4 (2)	C4—C3—C9—O4	5.3 (4)

### 1,3-Dimethyl 1-iodocyclohexa-3,5-diene-1,3-dicarboxylate (I) Hydrogen-bond geometry (Å, º)

**Table:**

*D*—H···*A*	*D*—H	H···*A*	*D*···*A*	*D*—H···*A*
C8—H8*A*···O1^i^	0.98	2.55	3.257 (4)	129

Symmetry code: (i) −*x*+1, −*y*+1, *z*−1/2.

### 1,3-Dimethyl 1-ethynylcyclohexa-3,5-diene-1,3-dicarboxylate (II) Crystal data

**Table:**

C_12_H_10_O_4_	*D*_x_ = 1.321 Mg m^−^^3^
*M**_r_* = 218.20	Mo *K*α radiation, λ = 0.71073 Å
Orthorhombic, *P**n**m**a*	Cell parameters from 2950 reflections
*a* = 10.1206 (5) Å	θ = 2.4–23.1°
*b* = 6.6219 (4) Å	µ = 0.10 mm^−^^1^
*c* = 16.3658 (8) Å	*T* = 223 K
*V* = 1096.80 (10) Å^3^	Column, colourless
*Z* = 4	0.54 × 0.12 × 0.10 mm
*F*(000) = 456	

### 1,3-Dimethyl 1-ethynylcyclohexa-3,5-diene-1,3-dicarboxylate (II) Data collection

**Table:**

Bruker APEXII CCD area detector diffractometer	932 reflections with *I* > 2σ(*I*)
φ and ω scans	*R*_int_ = 0.033
Absorption correction: multi-scan (SADABS; Sheldrick, 2008a)	θ_max_ = 27.0°, θ_min_ = 2.5°
*T*_min_ = 0.948, *T*_max_ = 0.990	*h* = −12→12
12397 measured reflections	*k* = −8→5
1292 independent reflections	*l* = −20→19

### 1,3-Dimethyl 1-ethynylcyclohexa-3,5-diene-1,3-dicarboxylate (II) Refinement

**Table:**

Refinement on *F*^2^	0 restraints
Least-squares matrix: full	Hydrogen site location: inferred from neighbouring sites
*R*[*F*^2^ > 2σ(*F*^2^)] = 0.039	H-atom parameters constrained
*wR*(*F*^2^) = 0.110	*w* = 1/[σ^2^(*F*_o_^2^) + (0.0486*P*)^2^ + 0.2932*P*] where *P* = (*F*_o_^2^ + 2*F*_c_^2^)/3
*S* = 1.03	(Δ/σ)_max_ < 0.001
1292 reflections	Δρ_max_ = 0.17 e Å^−^^3^
87 parameters	Δρ_min_ = −0.18 e Å^−^^3^

### 1,3-Dimethyl 1-ethynylcyclohexa-3,5-diene-1,3-dicarboxylate (II) Special details

**Table:**

Geometry. All esds (except the esd in the dihedral angle between two l.s. planes) are estimated using the full covariance matrix. The cell esds are taken into account individually in the estimation of esds in distances, angles and torsion angles; correlations between esds in cell parameters are only used when they are defined by crystal symmetry. An approximate (isotropic) treatment of cell esds is used for estimating esds involving l.s. planes.

### 1,3-Dimethyl 1-ethynylcyclohexa-3,5-diene-1,3-dicarboxylate (II) Fractional atomic coordinates and isotropic or equivalent isotropic displacement parameters (Å^2^)

**Table:**

	*x*	*y*	*z*	*U*_iso_*/*U*_eq_	Occ. (<1)
O1	1.25864 (13)	0.2500	0.40733 (10)	0.0552 (4)	
O2	1.08848 (14)	0.2500	0.32081 (9)	0.0519 (4)	
O3	1.15356 (16)	0.2500	0.70802 (10)	0.0759 (6)	
O4	0.94247 (15)	0.2500	0.74402 (9)	0.0617 (5)	
C1	0.98919 (18)	0.2500	0.60451 (11)	0.0339 (4)	
C2	1.08100 (18)	0.2500	0.54143 (12)	0.0350 (4)	
H2	1.1718	0.2500	0.5536	0.042*	
C3	1.03958 (18)	0.2500	0.46063 (12)	0.0339 (4)	
C4	0.90505 (19)	0.2500	0.44325 (12)	0.0361 (4)	
H4	0.8767	0.2500	0.3886	0.043*	
C5	0.81192 (17)	0.2500	0.50582 (12)	0.0348 (4)	
C6	0.85496 (18)	0.2500	0.58682 (12)	0.0340 (4)	
H6	0.7929	0.2500	0.6296	0.041*	
C7	1.0392 (2)	0.2500	0.68995 (13)	0.0428 (5)	
C8	0.9818 (3)	0.2500	0.82935 (14)	0.0791 (9)	
H8A	1.0203	0.1202	0.8431	0.119*	0.5
H8B	0.9050	0.2740	0.8634	0.119*	0.5
H8C	1.0465	0.3558	0.8385	0.119*	0.5
C9	1.1418	0.2500	0.3949	0.039	
C10	1.1813	0.2500	0.2530	0.067	
H10A	1.1353	0.2149	0.2030	0.101*	0.5
H10B	1.2505	0.1519	0.2633	0.101*	0.5
H10C	1.2202	0.3832	0.2474	0.101*	0.5
C11	0.67260 (19)	0.2500	0.48649 (12)	0.0406 (5)	
C12	0.5604 (2)	0.2500	0.47022 (14)	0.0532 (6)	
H12	0.4700	0.2500	0.4571	0.064*	

### 1,3-Dimethyl 1-ethynylcyclohexa-3,5-diene-1,3-dicarboxylate (II) Atomic displacement parameters (Å^2^)

**Table:**

	*U*^11^	*U*^22^	*U*^33^	*U*^12^	*U*^13^	*U*^23^
O1	0.0293 (8)	0.0828 (11)	0.0536 (10)	0.000	0.0049 (7)	0.000
O2	0.0409 (9)	0.0783 (11)	0.0364 (8)	0.000	0.0066 (7)	0.000
O3	0.0329 (9)	0.1475 (18)	0.0473 (10)	0.000	−0.0079 (8)	0.000
O4	0.0362 (9)	0.1153 (14)	0.0336 (8)	0.000	−0.0026 (7)	0.000
C1	0.0294 (10)	0.0371 (10)	0.0352 (11)	0.000	−0.0006 (8)	0.000
C2	0.0257 (9)	0.0385 (10)	0.0408 (11)	0.000	−0.0029 (8)	0.000
C3	0.0295 (10)	0.0338 (9)	0.0385 (11)	0.000	0.0023 (8)	0.000
C4	0.0343 (11)	0.0404 (10)	0.0334 (10)	0.000	−0.0020 (9)	0.000
C5	0.0275 (9)	0.0379 (10)	0.0389 (11)	0.000	−0.0002 (8)	0.000
C6	0.0276 (9)	0.0398 (10)	0.0347 (11)	0.000	0.0011 (8)	0.000
C7	0.0299 (11)	0.0568 (12)	0.0416 (12)	0.000	−0.0013 (9)	0.000
C8	0.0550 (16)	0.151 (3)	0.0315 (13)	0.000	−0.0049 (12)	0.000
C9	0.034	0.043	0.039	0.000	0.003	0.000
C10	0.063	0.098	0.041	0.000	0.018	0.000
C11	0.0341 (11)	0.0558 (12)	0.0319 (10)	0.000	0.0003 (9)	0.000
C12	0.0340 (12)	0.0849 (17)	0.0409 (13)	0.000	−0.0041 (10)	0.000

### 1,3-Dimethyl 1-ethynylcyclohexa-3,5-diene-1,3-dicarboxylate (II) Geometric parameters (Å, º)

**Table:**

O1—C9	1.1998 (14)	C4—C5	1.392 (3)
O2—C9	1.3272 (15)	C4—H4	0.9400
O2—C10	1.4544 (14)	C5—C6	1.395 (3)
O3—C7	1.195 (3)	C5—C11	1.445 (3)
O4—C7	1.319 (3)	C6—H6	0.9400
O4—C8	1.452 (3)	C8—H8A	0.9700
C1—C2	1.389 (3)	C8—H8B	0.9700
C1—C6	1.389 (3)	C8—H8C	0.9700
C1—C7	1.487 (3)	C10—H10A	0.9700
C2—C3	1.387 (3)	C10—H10B	0.9700
C2—H2	0.9400	C10—H10C	0.9700
C3—C4	1.391 (3)	C11—C12	1.166 (3)
C3—C9	1.4925 (18)	C12—H12	0.9400
			
C9—O2—C10	115.75 (10)	O3—C7—O4	123.6 (2)
C7—O4—C8	116.19 (18)	O3—C7—C1	124.21 (19)
C2—C1—C6	119.96 (18)	O4—C7—C1	112.23 (17)
C2—C1—C7	118.13 (17)	O4—C8—H8A	109.5
C6—C1—C7	121.91 (17)	O4—C8—H8B	109.5
C3—C2—C1	120.42 (17)	H8A—C8—H8B	109.5
C3—C2—H2	119.8	O4—C8—H8C	109.5
C1—C2—H2	119.8	H8A—C8—H8C	109.5
C2—C3—C4	119.39 (18)	H8B—C8—H8C	109.5
C2—C3—C9	118.53 (15)	O1—C9—O2	123.76 (10)
C4—C3—C9	122.09 (16)	O1—C9—C3	124.12 (11)
C3—C4—C5	120.83 (18)	O2—C9—C3	112.12 (9)
C3—C4—H4	119.6	O2—C10—H10A	109.5
C5—C4—H4	119.6	O2—C10—H10B	109.5
C4—C5—C6	119.18 (17)	H10A—C10—H10B	109.5
C4—C5—C11	119.98 (18)	O2—C10—H10C	109.5
C6—C5—C11	120.84 (17)	H10A—C10—H10C	109.5
C1—C6—C5	120.21 (17)	H10B—C10—H10C	109.5
C1—C6—H6	119.9	C12—C11—C5	179.5 (2)
C5—C6—H6	119.9	C11—C12—H12	180.0
			
C6—C1—C2—C3	0.000 (1)	C8—O4—C7—O3	0.000 (1)
C7—C1—C2—C3	180.000 (1)	C8—O4—C7—C1	180.000 (1)
C1—C2—C3—C4	0.000 (1)	C2—C1—C7—O3	0.000 (1)
C1—C2—C3—C9	180.000 (1)	C6—C1—C7—O3	180.000 (1)
C2—C3—C4—C5	0.000 (1)	C2—C1—C7—O4	180.000 (1)
C9—C3—C4—C5	180.000 (1)	C6—C1—C7—O4	0.000 (1)
C3—C4—C5—C6	0.000 (1)	C10—O2—C9—O1	0.000 (1)
C3—C4—C5—C11	180.000 (1)	C10—O2—C9—C3	180.000 (1)
C2—C1—C6—C5	0.000 (1)	C2—C3—C9—O1	0.000 (1)
C7—C1—C6—C5	180.000 (1)	C4—C3—C9—O1	180.000 (1)
C4—C5—C6—C1	0.000 (1)	C2—C3—C9—O2	180.000 (1)
C11—C5—C6—C1	180.000 (1)	C4—C3—C9—O2	0.000 (1)

### 1,3-Dimethyl 1-ethynylcyclohexa-3,5-diene-1,3-dicarboxylate (II) Hydrogen-bond geometry (Å, º)

**Table:**

*D*—H···*A*	*D*—H	H···*A*	*D*···*A*	*D*—H···*A*
C12—H12···O1^i^	0.94	2.29	3.223 (1)	172

Symmetry code: (i) *x*−1, *y*, *z*.
